# Unilateral bifid ureter

**DOI:** 10.11604/pamj.2016.25.204.9529

**Published:** 2016-11-29

**Authors:** Souhail Regragui, Amine Slaoui

**Affiliations:** 1 University Mohammed V, Urology B Ibn Sina Hospital, Rabat, Morocco

**Keywords:** Ureter, bifidity, duplex kidney

## Image in medicine

A bifid ureter is a congenital renal tract abnormality due to some error or disturbance during development. It is an example of incomplete duplication of urinary collecting system. A bifid ureter is formed when a duplex kidney drain into separate ureters that unite before attending the bladder. Bifid ureter is reported to be twice more common in females and on the right side. The majority of the investigators have reported this anomaly in association with other disease conditions. Nevertheless, some complications have been reported such as: frequent urinary tract infection, uretero ureteric reflux, ureteric stenosis, urinary lithiasis, and pyelonephritis, non-functioning of kidney units. We report a case of left bifid ureter on a male patient. A male patient of 45 YO without significant antecedent consulted for dysuria. An intravenous urography objectified urethral stenosis and left ureter bifidity, which has been discovered incidentally. The kidney function was normal and the urine culture was positive: E. Coli. The patient underwent endoscopic internal urethrotomy, and the therapeutic strategy for the bifid ureter was the abstention and closer survey.

**Figure 1:**
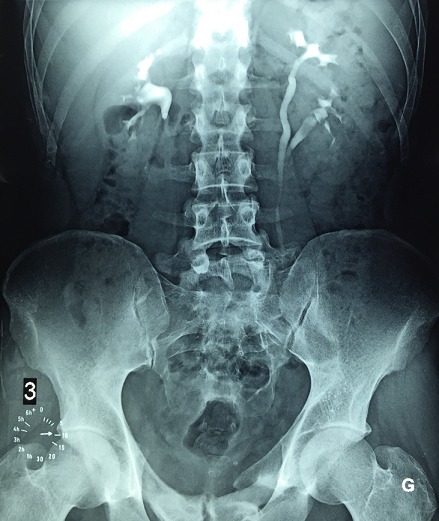
Left bifid ureter

